# AI-derived cardiothoracic structures and coronary artery calcification volumes on non-contrast CT as predictors of mortality in pulmonary hypertension

**DOI:** 10.1007/s44449-026-00005-2

**Published:** 2026-11-04

**Authors:** Turki Nasser Alnasser, Alireza Hokmabadi, Michael Sharkey, David G. Kiely, Samer Alabed, Andrew J. Swift

**Affiliations:** 1 School of Medicine and Population Health, The University of Sheffield, Sheffield, UK; 2 Radiological Sciences Department, College of Applied Medical Science, King Saud bin Abdulaziz University for Health Science, Riyadh, Saudi Arabia; 3 King Abdullah International Medical Research Centre (KAIMRC), Riyadh, Saudi Arabia; 4 Insigneo Institute, Faculty of Engineering, The University of Sheffield, Sheffield, UK; 5 National Institute for Health and Care Research, Sheffield Biomedical Research Centre, Sheffield, UK; 6 Sheffield Pulmonary Vascular Disease Unit, Sheffield Teaching Hospitals NHS Trust, Sheffield, UK; 7 Department of Clinical Radiology, Sheffield Teaching Hospitals, Sheffield, UK

**Keywords:** Non-contrast CT, Pulmonary hypertension, Prognostic, AI, Coronary

## Abstract

**Objective:**

To assess the prognostic value of AI-derived cardiothoracic structures and coronary artery calcification volumetric measurements in pulmonary hypertension (PH) using non-gated, non-contrast CT.

**Materials and methods:**

Patients with suspected PH who underwent non-gated, non-contrast chest CT and right heart catheterisation within one month were retrospectively identified from the Assessing the Spectrum of PH Identified at a Referral Centre (ASPIRE) registry. Two validated deep learning models were used to segment and measure the volumes of cardiothoracic structures and coronary artery calcifications. The prognostic value of AI-derived cardiothoracic structures and coronary artery calcification volumetric measurements was assessed using univariable and multivariable Cox regression adjusted for demographics and PH subgroups and REVEAL scores.

**Results:**

Six hundred twenty patients were included (mean age 64 ± 13 years; 62% female; 388 patients died during follow-up), comprising 202 patients with pulmonary arterial hypertension, 84 with PH-LHD, 112 with PH associated with lung disease, 65 with chronic thromboembolic PH, 9 with unclear PH, and 148 patients without PH. Calcifications were present in the right coronary artery (RCA), mainstem and left anterior descending artery (LAD), and left circumflex artery (LCX) in 45%, 67%, and 35% of patients, respectively. In multivariable Cox regression analysis adjusted for age, sex, and PH subgroup, right atrial (RA) volume was independently associated with mortality (HR 1.024, 95% CI: 1.009–1.039, *p* = 0.001), as were coronary artery calcifications in the RCA (HR 1.378, 95% CI: 1.113–1.705, *p* = 0.003), mainstem and LAD (HR 1.868, 95% CI: 1.441–2.421, *p* < 0.001), and LCX (HR 1.342, 95% CI: 1.084–1.660, *p* = 0.007). After further adjustment for the REVEAL risk score, these associations remained significant (RA volume HR 1.026, RCA calcification HR 1.604, mainstem and LAD calcification HR 2.339, LCX calcification HR 1.576; all *p* < 0.001).

**Conclusion:**

AI-derived RA volume and coronary artery calcifications are independently associated with increased mortality in PH patients.

**Key Points:**

**Question** *The prognostic significance of AI-derived cardiothoracic volumes and coronary artery calcification measured on routine non-gated, non-contrast chest CT in patients with PH remains uncertain.*

**Findings** *AI-derived RA volume and coronary artery calcification volumes independently predicted mortality in PH patients, even after adjustment for demographics, PH subgroups, and REVEAL score.*

**Relevance statement** *Automated CT-based AI measurements can provide additional prognostic biomarkers to improve risk stratification in PH beyond established clinical scores.*

**Graphical Abstract:**

**Figure:**
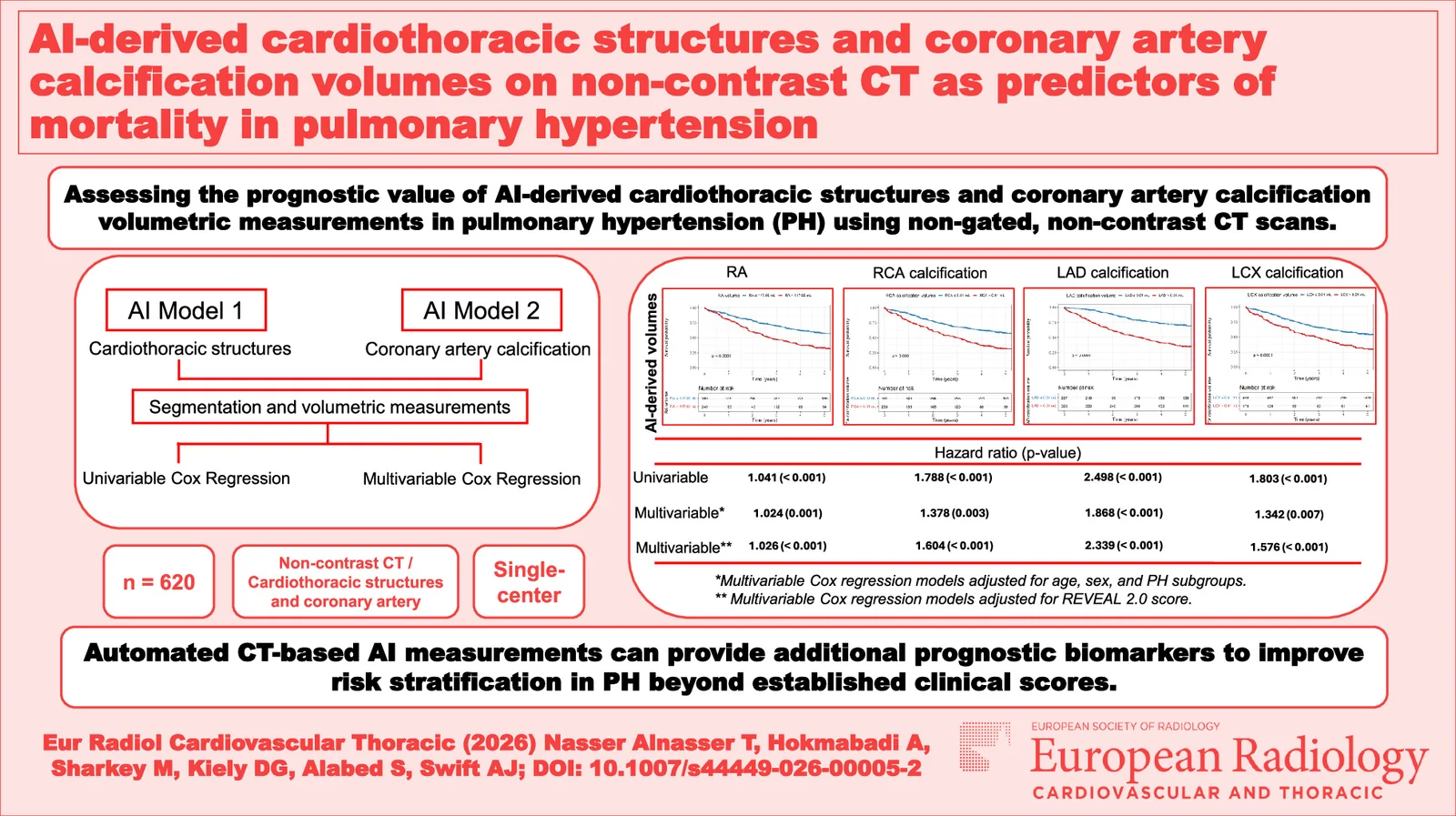

## Introduction

Pulmonary hypertension (PH) is a progressive and life-limiting disorder characterised by elevated mean pulmonary arterial pressure (mPAP) and increased pulmonary vascular resistance (PVR) [1, 2]. Despite advances in disease-targeted therapies and structured risk stratification frameworks, PH remains associated with substantial morbidity and mortality [2–5]. Furthermore, the burden of PH continues to increase, with growing numbers of patients requiring specialist assessment and follow-up [5–7].

Computed tomography (CT) is an important imaging modality in the PH assessment, providing detailed anatomical information for both diagnosis and prognostication [1, 8]. Dilatation of cardiothoracic structures, including the right atrium (RA), pulmonary artery (PA), the PA to ascending aorta ratio (PA/AAo), and left atrium (LA), is linked to worse survival in PH, highlighting the prognostic value of these anatomical markers [8–13].

Comorbidities are common in PH patients and can significantly influence prognosis, clinical management, and outcomes [2, 14]. Coronary artery calcification on CT is an independent prognostic marker associated with all-cause and cardiovascular mortality, even after adjustment for age and other comorbidities [14–16].

Quantitative CT measurements of cardiothoracic structures are typically performed manually, which is time-consuming and subject to inter-observer variability. With the growing use of artificial intelligence (AI), deep learning (DL) approaches show promise for automating these measurements, offering rapid, accurate, and reproducible quantification of cardiovascular structures [17–20].

This study aimed to evaluate the prognostic value of AI-derived volume measurements of cardiovascular structures and coronary artery calcification from non-gated, non-contrast CT in PH by assessing their association with mortality and prognostic significance after adjustment for demographic factors, PH subgroups, and established clinical risk scores.

## Methods

### Study population and included CT scans

Patients and their non-gated, non-contrast CT scans were identified retrospectively from the ASPIRE registry, which includes patients who underwent evaluation for suspected PH at the Sheffield Pulmonary Vascular Disease Unit (Sheffield, England) and had not received any prior treatment. Patients enrolled in the registry typically underwent specialist clinical assessment, pulmonary function testing, multimodal imaging, and right heart catheterisation (RHC) [21]. Eligibility for the present study required patients to be aged 18 years or older, have a suspected diagnosis of PH, and have undergone both RHC and CT imaging within one month of assessment. CT scans were included if they had a slice thickness of ≤ 5 mm, a field of view of 400 × 400 mm, and an acquisition matrix of 512 × 512. Scans were acquired using standard helical mode parameters, with typical settings including a pitch of approximately 1, a tube current ranging from 100–500 mA, and a tube voltage of 120 kV. Patients were excluded if RHC and non-contrast CT were not performed within the specified timeframe.

### Study flow and design

**Fig. 1:**
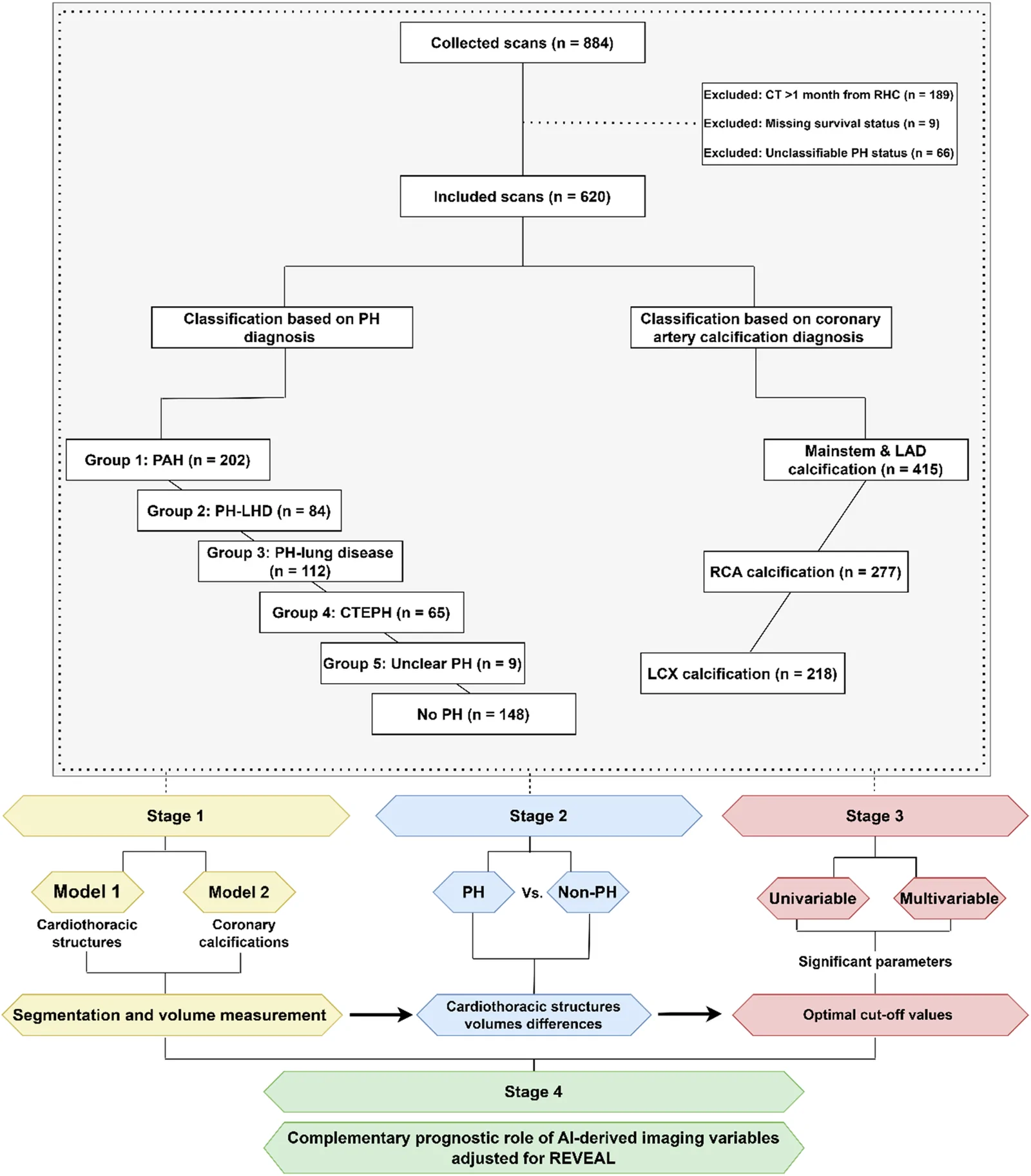
Flow chart of the study design. PH, pulmonary hypertension; PAH, pulmonary arterial hypertension; LHD, left heart disease; CTEPH, chronic thromboembolic pulmonary hypertension; RCA, right coronary artery; LAD, left anterior descending artery; LCX, left circumflex artery

This study was designed to evaluate the association between AI-derived cardiac structure volumes, coronary artery calcifications, and all-cause mortality in PH patients. In stage 1, cardiothoracic structure volumes, including the ventricles (i.e., combined right and left ventricles), RA, LA, PA, AAo, descending aorta (DAo), and coronary artery calcification presence and volumes, were derived using DL-based models. In stage 2, AI-derived cardiothoracic volumes were compared between PH and non-PH groups, with additional subgroup analyses performed to evaluate differences between PH subgroups and the non-PH group. In stage 3, the association between AI-derived cardiothoracic volumes and coronary artery calcification with all-cause mortality was assessed. Univariable Cox regression was performed for both cardiac structure volumes and the presence of coronary artery calcification. Multivariable Cox regression further evaluated these variables, adjusting for age, sex, and PH subgroups. For parameters showing significant associations in both analyses, optimal cutoff values were determined and subsequently dichotomised to assess mortality risk above these thresholds. In Stage 4, the AI-derived imaging variables' prognostic significance was evaluated after adjustment for established clinical risk scores. Registry to Evaluate Early and Long-Term PAH Disease Management (REVEAL) is a risk stratification tool employed in pulmonary arterial hypertension (PAH). The score is calculated using a combination of demographic, clinical, functional, exercise, laboratory, imaging, and haemodynamic parameters, including factors such as age, PAH aetiology, World Health Organisation functional class, 6-min walk distance, and RHC measurements, to estimate mortality risk and disease severity [22] (Fig. 1).

### Stage 1: segmentation models development

The cardiothoracic segmentation DL model used in this study was developed and validated in earlier work [20]. In summary, the model was developed using the nnU-Net framework [23], which provides a self-configuring baseline for medical image segmentation. The model was trained on manual annotations of multi-cardiothoracic structures from 50 non-contrast CT scans. Its performance was evaluated on an external cohort of 55 scans and compared with previously reported segmentation results. The model was able to segment the right and left ventricles (RV and LV), RA, LA, PA, Aao, and DAo, with high performance (i.e., Dice similarity coefficient (DSC) ≥ 0.87 and Hausdorff Distance ≤ 1.7). Diagnostic accuracy was further assessed in 368 scans, achieving AUCs of 0.84–0.88, sensitivities of 70–94%, and specificities of 63–100%.

The coronary artery calcification detection model was developed and validated in earlier work [24]. The model was developed using 69 scans to segment the right coronary artery (RCA), mainstem and left anterior descending coronary artery (LAD), left circumflex coronary artery (LCX) and detect the calcifications. The model was visually assessed with 100 scans by two experienced observers and tested on 473 scans. The calcification detection model demonstrated high diagnostic accuracy, with a sensitivity of 95%, specificity of 98%, positive predictive value of 99%, and negative predictive value of 88% compared to radiologists’ reports.

These two DL models were applied in this study to segment and quantify the volumes of cardiac chambers (i.e., RV, LV, RA, and LA) and great vessels (i.e., PA, AAo, and DAo) and coronary artery calcifications in RCA, mainstem and LAD, and LCX.

### Statistical analysis

Continuous variables are presented as mean ± standard deviation (SD). AI-derived cardiac structure volumes were compared between patients with PH and those without PH using independent samples *t*-tests. Subgroup analyses were additionally performed to compare PH subgroups against the non-PH group. Homogeneity of variance was assessed with Levene’s test, and the appropriate *t*-test (equal or unequal variances assumed) was applied accordingly. Standardised effect sizes were calculated using Cohen’s *d*, Hedges’ correction, and Glass’s delta to quantify the magnitude of differences. Results are presented as mean ± SD with corresponding *p*-values (Stage 2).

To assess the association between AI-derived cardiac structure volumes and the presence of coronary artery calcification (binary variable) and all-cause mortality, univariable and multivariable Cox proportional hazards regression models were performed. In univariable analysis, each cardiac structure volumetric measurement was entered into a separate model as a continuous variable, including ventricles, RA, LA, PA, AAo, and DAo. For volumetric measures, hazard ratios (HRs) were calculated per 10-mL increase in volume. For the PA/AAo ratio and coronary artery calcification presence, HRs reflect the change in hazard per unit. In multivariable analysis, models were adjusted for age, sex, and PH subgroups. HR with 95% CI and *p*-values were reported.

For parameters showing significant associations in both analyses, optimal cutoff values were determined using receiver operating characteristic (ROC) curve analysis and the Youden index, where variables were subsequently dichotomised into low (≤ cutoff) and high (> cutoff) groups. Multivariable Cox regression analysis adjusted for age, sex, and PH subgroups was then performed using these dichotomised variables. Adjusted HRs, 95% CIs, and *p*-values are presented. These models quantify the risk of mortality associated with values above the Youden-derived threshold. Survival time was defined as the interval (in days) from the scan date to death, or last follow-up, and overall death was coded as a binary variable (0 = alive, 1 = deceased). Kaplan–Meier survival curves were created and compared using the log-rank test, and the number of patients at risk was reported at specified time points (Stage 3).

The ability of the AI-derived cardiovascular and coronary artery calcification to improve risk discrimination and prognostic accuracy beyond the established risk scoring system was evaluated using multivariable Cox regression analysis adjusted for REVEAL 2.0 (Stage 4).

Statistical analyses were performed using SPSS Statistics for Windows (Version 29.0, IBM Corp, 2022) and R (Version 4.5.1). All tests were two-sided, and a *p*-value < 0.05 was considered statistically significant.

## Results

### Patient characteristics

The demographics of the included patients are summarised in Table 1 and Fig. 1. A total of 884 patients were initially identified from the ASPIRE registry. Of these, 264 were excluded because 189 had a CT scan performed more than one month from RHC, 9 had missing survival status, and 66 were unclassifiable (i.e., missing RHC haemodynamic and PH subtypes). The final study cohort consisted of 620 patients enrolled between 2006 and 2020 (62% female, mean age 64 ± 13 years). The majority of included patients had PH (76%), including 202 patients diagnosed with PAH (Group 1), 84 patients diagnosed with PH due to Left Heart Disease (PH-LHD) (Group 2), 112 patients diagnosed with PH due to lung disease (Group 3), 65 patients diagnosed with chronic thromboembolic pulmonary hypertension (CTEPH) (Group 4), 9 patients diagnosed with unclear PH (Group 5), and 148 patients were non-PH patients. Among these patients, 277 patients had calcifications in RCA (45%), 415 patients had calcifications in mainstem and LAD (67%), and 218 patients had calcifications in LCX (35%). Out of 620 patients included in the study, 388 died (63%). The survival duration ranged from 2 to 5187 days, with a mean survival of 1,659 ± 1,207 days. The majority of included patients were white British according to documented self-reported ethnicity (80%). CT scans were performed at Sheffield, England, using systems by GE Healthcare (78%), Canon (18%) and Siemens (4%).

**Table 1:** Demographic information of the patient population

		Included patients (*n* = 620)
Age (years ± SD)		64 ± 13
Gender	Male	237 (38.0%)
	Female	383 (62.0%)
Ethnicity	White	497 (80.2%)
	Non-White	43 (6.9%)
	Not stated	80 (12.9%)
Scanner manufacturers	GE LightSpeed Pro 32	263 (42.4%)
	GE LightSpeed VCT	221 (35.7%)
	Canon Aquilion ONE	109 (17.6%)
	Siemens Sensation	15 (2.4%)
	Siemens SOMATOM Definition	12 (1.9%)
Scan dates		2006–2020
CT image slices (mean ± SD)		241 ± 91
Diagnosis	Group 1: PAH	202 (32.6%)
	Group 2: PH-LHD	84 (13.6%)
	Group 3: PH-lung disease	112 (18.1%)
	Group 4: CTEPH	65 (10.5%)
	Group 5: Unclear PH	9 (1.3%)
	No PH	148 (23.9%)
Coronary calcification	RCA	277 (44.7%)
	Mainstem + LAD	415 (66.9%)
	LCX	218 (35.2%)
	In all three vessels^*^	167 (26.9%)
	In any vessel^**^	447 (72.1%)
Mortality (%)	1-year mortality	92 (14.8%)
	2-year mortality	180 (29.0%)
	5-year mortality	317 (51.1%)
	Overall mortality	388 (62.6%)
Survival time (in days)	Range	2–5187
	Mean ± SD	1659 ± 1207

*PAH* pulmonary arterial hypertension, *PH* pulmonary hypertension, *LHD* left heart disease, *CTEPH* chronic thromboembolic pulmonary hypertension, *RCA* right coronary artery, *LAD* left anterior descending artery, *LCX* left circumflex artery

^*^ Calcifications in all three segmented arteries

^**^ Calcification in any segmented artery

### AI-Derived cardiothoracic structures volumes in PH and non-PH

The full results of the comparison of AI-derived cardiothoracic structure volumes between PH (and PH subgroups) vs non-PH patients are summarised in Table 2.

**Table 2:** Comparison of AI-derived cardiac structure and coronary calcification volumes between PH (and PH subgroups) vs no PH using independent *t*-tests

PH subgroup	Volumes	PH/PH subgroups (mean ± SD)	No PH (mean ± SD) (*n* = 148)	Mean difference in mL (95% CI)	*p*-value	Sig	Cohen’s *d*
PH (*n* = 472)	Ventricles	373.2 ± 166.5	321.8 ± 117.3	51.4 (22.5–80.3)	**< 0.001**	*	0.33
	LA	74.4 ± 44.5	68.4 ± 38.3	6.0 (−2.0 to 13.9)	0.112		0.14
	RA	123.6 ± 74.6	79.3 ± 51.7	44.3 (31.4–57.2)	**< 0.001**	*	0.63
	AAo	112.9 ± 45.7	109.3 ± 46.8	3.6 (−4.9 to 12.1)	0.408		0.08
	PA	120.7 ± 56.1	84.1 ± 38.1	36.6 (26.9–46.3)	**< 0.001**	*	0.70
	DAo	108.8 ± 41.2	104.5 ± 39.7	4.2 (−3.3 to 11.8)	0.265		0.10
	Total calcifications	0.219 ± 0.307	0.155 ± 0.252	0.064 (−0.113 to −0.015)	0.011	*	0.22
Group 1: PAH (*n* = 202)	Ventricles	360.6 ± 159.2	321.8 ± 117.3	38.8 (8.36–69.24)	**0.005**	*	0.27
	LA	62.4 ± 35.3	68.4 ± 38.3	−6.03 (−1.86 to 13.91)	0.134		0.17
	RA	121.5 ± 72.6	79.3 ± 51.7	42.2 (29.09–55.23)	**< 0.001**	*	0.65
	AAo	102.4 ± 43.3	109.3 ± 46.8	−6.92 (−2.74 to 16.59)	0.16		0.15
	PA	122.4 ± 67.3	84.1 ± 38.1	38.3 (27.15–49.47)	**< 0.001**	*	0.67
	DAo	97.7 ± 36.2	104.5 ± 39.7	−6.90 (−1.24 to 15.03)	0.096		0.18
	Total Calcifications	0.150 ± 0.264	0.155 ± 0.252	−0.005 (−0.049 to 0.06)	0.849		−0.02
Group 2: PH-LHD (*n* = 84)	Ventricles	444.3 ± 176.3	321.8 ± 117.3	122.5 (84.42–160.58)	**< 0.001**	*	0.87
	LA	128.2 ± 45.4	68.4 ± 38.3	59.9 (48.27–71.46)	**< 0.001**	*	1.46
	RA	168.0 ± 80.1	79.3 ± 51.7	88.7 (69.43 - 107.92)	**< 0.001**	*	1.40
	AAo	127.4 ± 45.2	109.3 ± 46.8	18.1 (5.64–30.44)	**0.004**	*	0.39
	PA	121.2 ± 39.6	84.1 ± 38.1	37.1 (26.59–47.66)	**< 0.001**	*	0.96
	DAo	127.1 ± 43.1	104.5 ± 39.7	22.6 (11.31–33.90)	**< 0.001**	*	0.55
	Total calcifications	0.333 ± 0.341	0.155 ± 0.252	0.178 (0.094–0.262)	**< 0.001**	*	0.62
Group 3: PH-lung disease (*n* = 112)	Ventricles	361.7 ± 143.3	321.8 ± 117.3	39.9 (7.14–72.68)	**0.009**	*	0.31
	LA	62.8 ± 30.2	68.4 ± 38.3	−5.60 (−2.76 to 13.96)	0.189		0.16
	RA	103.5 ± 65.3	79.3 ± 51.7	24.2 (9.46–39.01)	**0.001**	*	0.42
	AAo	117.0 ± 39.5	109.3 ± 46.8	7.71 (−2.85 to 18.26)	0.152		0.18
	PA	121.3 ± 47.3	84.1 ± 38.1	37.1 (26.38–47.89)	**< 0.001**	*	0.88
	DAo	113.7 ± 42.7	104.5 ± 39.7	9.14 (−1.08 to 19.37)	0.079		0.22
	Total calcifications	0.253 ± 0.296	0.155 ± 0.252	0.098 (0.03–0.167)	**0.005**	*	0.36
Group 4: CTEPH (*n* = 65)	Ventricles	345.9 ± 189.2	321.8 ± 117.3	24.1 (−7.1 to 66.0)	0.346		0.17
	LA	63.2 ± 40.5	68.4 ± 38.3	5.1 (−6.6 to 16.9)	0.387		0.13
	RA	108.4 ± 69.0	79.3 ± 51.7	29.1 (10.2 to 48.1)	**0.003**	*	0.51
	AAo	117.2 ± 56.8	109.3 ± 46.8	−7.9 (−23.9 to 6.8)	0.326		0.16
	PA	112.2 ± 53.2	84.1 ± 38.1	28.1 (13.6–42.6)	**< 0.001**	*	0.65
	DAo	109.8 ± 40.1	104.5 ± 39.7	5.2 (−11.3 to 16.9)	0.381		0.13
	Total calcifications	0.196 ± 0.326	0.155 ± 0.252	0.042 (−0.049 to 0.132)	**0.363**		0.15

*PH* pulmonary hypertension, *CI* confidence interval, *PAH* pulmonary arterial hypertension, *LHD* left heart disease, *CTEPH* chronic thromboembolic pulmonary hypertension, *LA* left atrium, *RA* right atrium, *AAo* ascending aorta, *PA* pulmonary artery, *DAo* descending aorta, *mL* millilitre

Statistically significant *p*-values are in bold

#### PH vs non-PH

Patients with PH demonstrated significantly larger RA, ventricular, and PA volumes compared with the non-PH group (mean differences: RA 44.3 mL; ventricular 51.4 mL; PA 36.6 mL; all *p* < 0.001), with no significant differences observed in LA, AAo, or DAo.

#### PAH vs non-PH

Patients with PAH demonstrated significantly larger RA, ventricular, and PA volumes compared with the non-PH group (mean differences: RA 42.2 mL, *p* < 0.001; ventricular 38.8 mL, *p* = 0.005; PA 38.3 mL, *p* < 0.001). In contrast, LA, AAo, and DAo volumes were higher in the non-PH group; however, these differences did not reach statistical significance.

#### PH-LHD vs non-PH

Patients with PH-LHD demonstrated significantly larger RA, LA, ventricular, and vascular volumes compared with the non-PH group (mean differences: RA 88.7 mL, *p* < 0.001; LA 59.9 mL, *p* < 0.001; ventricular 122.5 mL, *p* < 0.001; AAo 18.1 mL, *p* = 0.004; PA 37.1 mL, *p* < 0.001; DAo 22.6 mL, *p* < 0.001).

#### PH-lung disease vs non-PH

Patients with PH associated with lung disease demonstrated significantly larger RA, ventricular, and PA volumes compared with the non-PH group (mean differences: RA 24.2 mL, *p* = 0.001; ventricular 39.9 mL, *p* = 0.009; PA 37.1 mL, *p* < 0.001). However, LA, AAo, and DAo volumes did not differ significantly between groups.

#### CTEPH vs non-PH

Patients with CTEPH demonstrated significantly larger RA and PA volumes compared with the non-PH group (mean differences: RA 29.1 mL, *p* = 0.003; PA 28.1 mL, *p* < 0.001), with no significant differences observed in ventricles, LA, AAo, or DAo volumes.

### Association of cardiothoracic structures volumes and coronary artery calcification with mortality

Table 3 summarises the univariable and multivariable Cox regression analyses examining the association between AI-derived cardiovascular structure volumes, coronary artery calcifications, and all-cause mortality.

**Table 3:** Univariable and multivariable Cox regression analysis of AI-derived structure volumes and mortality in PH

Structure	Univariable analysis				Multivariable analysis^**^				Multivariable analysis^***^			
	HR^+^	95% CI	*p*-value	Sig	HR^+^	95% CI	*p*-value	Sig	HR^+^	95% CI	*p*-value	Sig
Ventricles	1.002	0.996–1.009	0.508		1.001	0.994–1.009	0.707		0.995	0.988–1.002	0.152	
LA	1.045	1.022–1.068	< 0.001	*	1.003	0.979–1.028	0.788		1.024	1.001–1.047	0.043	*
RA	1.041	1.027–1.055	< 0.001	*	1.024	1.009–1.039	0.001	*	1.026	1.012–1.041	< 0.001	*
AAo	1.024	1.001–1.047	0.040	*	0.987	0.962–1.012	0.313		0.999	0.976–1.023	0.931	
PA	1.025	1.007–1.043	0.008	*	1.010	0.989–1.032	0.340		1.005	0.985–1.025	0.655	
DAo	1.043	1.016–1.070	0.002	*	0.999	0.968–1.031	0.941		1.010	0.982–1.038	0.493	
PA/AAo > 1	1.199	0.980–1.467	0.077		1.356	1.090–1.686	0.006	*	1.153	0.942–1.411	0.166	
RCA calcification	1.788	1.462–2.184	< 0.001	*	1.378	1.113–1.705	0.003	*	1.604	1.311–1.964	< 0.001	*
Mainstem + LAD calcification	2.498	1.959–3.182	< 0.001	*	1.868	1.441–2.421	< 0.001	*	2.339	1.834–2.983	< 0.001	*
LCX calcification	1.803	1.472–2.206	< 0.001	*	1.342	1.084–1.660	0.007	*	1.576	1.285–1.933	< 0.001	*
Calcification in any coronary artery	2.415	1.865–3.127	< 0.001	*	2.191	1.670–2.874	< 0.001	*	2.299	1.775–2.979	< 0.001	*
Calcification in all coronary arteries	1.882	1.524–2.324	< 0.001	*	1.673	1.347–2.077	< 0.001	*	1.661	1.342–2.055	< 0.001	*

*LA* left atrium, *RA* right atrium, *AAo* ascending aorta, *PA* pulmonary artery, *DAo* descending aorta, *RCA* right coronary artery, *LAD* left anterior descending artery, *LCX* left circumflex artery, *HR* hazard ratio, *CI* confidence interval

^*^ Significant

^**^ Multivariable Cox regression models adjusted for age, sex, and PH subgroups

^***^ Multivariable Cox regression models adjusted for REVEAL 2.0 as exploratory analysis. +HRs indicate hazard per 10-mL increase in AI-derived volume from non-contrast CT

#### Univariable analysis

In univariable analyses, larger LA volume (HR 1.045, 95% CI: 1.022–1.068; *p* < 0.001), RA volume (HR 1.041, 95% CI: 1.027–1.055; *p* < 0.001), AAo volume (HR 1.024, 95% CI: 1.001–1.047; *p* = 0.040), PA volume (HR 1.025, 95% CI: 1.007–1.043; *p* = 0.008), DAo volume (HR 1.043, 95% CI: 1.016–1.070; *p* = 0.002), RCA calcification (HR 1.788, 95% CI: 1.462–2.184; *p* < 0.001), mainstem and LAD calcification (HR 2.498, 95% CI: 1.959–3.182; *p* < 0.001), and LCX calcification (HR 1.803, 95% CI: 1.472–2.206; *p* < 0.001) were all significantly associated with increased mortality. In contrast, ventricular volume and the PA/AAo > 1 were not significant predictors in univariable models.

#### Multivariable analysis adjusted for age, sex, and PH subgroups

RA volume remained independently associated with mortality (HR 1.024, 95% CI: 1.009–1.039; *p* = 0.001). Coronary artery calcification also remained significant across all segmented arteries: RCA (HR 1.378, 95% CI: 1.113–1.705; *p* = 0.003), mainstem and LAD (HR 1.868, 95% CI: 1.441–2.421; *p* < 0.001), and LCX (HR 1.342, 95% CI: 1.084–1.660; *p* = 0.007). The PA/AAo > 1 demonstrated a strong independent association with mortality (HR 1.361, 95% CI: 1.094–1.694; *p* = 0.006). On the other hand, PA, LA, AAo, DAo, and ventricular volumes were not independently and significantly associated with mortality after adjustments.

#### Multivariable analysis adjusted for REVEAL 2.0 (exploratory analysis in PH)

LA and RA volumes were significantly associated with increased mortality risk. Specifically, each 10-ml increase in LA volume was associated with a modest rise in risk (HR 1.024, 95% CI: 1.001–1.047, *p* = 0.043), while RA volume demonstrated a slightly stronger association (HR 1.026, 95% CI: 1.012–1.041, *p* < 0.001). Coronary artery calcification was significantly associated with mortality across all evaluated vessels. RCA calcification was associated with an increased risk of mortality (HR 1.604, 95% CI: 1.311–1.964), with an even stronger association observed for mainstem and LAD calcification (HR 2.339, 95% CI: 1.834–2.983). LCX calcification was also significantly associated with mortality (HR 1.576, 95% CI: 1.285–1.933) (all *p* < 0.001). In contrast, ventricles, AAo, PA, DAo, and PA/AAo ratio > 1.0 were not significantly associated with mortality.

### Optimal cutoff values and survival analysis for significant parameters

Significant predictors from the univariable and multivariable Cox regression analysis were subsequently categorised using optimal cut-off values derived from the Youden index. Multivariable Cox regression models adjusted for age, sex, and PH subgroups were then performed using these dichotomised variables. For the PA/AAo ratio, a threshold of > 1.0 was applied, reflecting an established clinically validated cut-off [1].

**Fig. 2:**
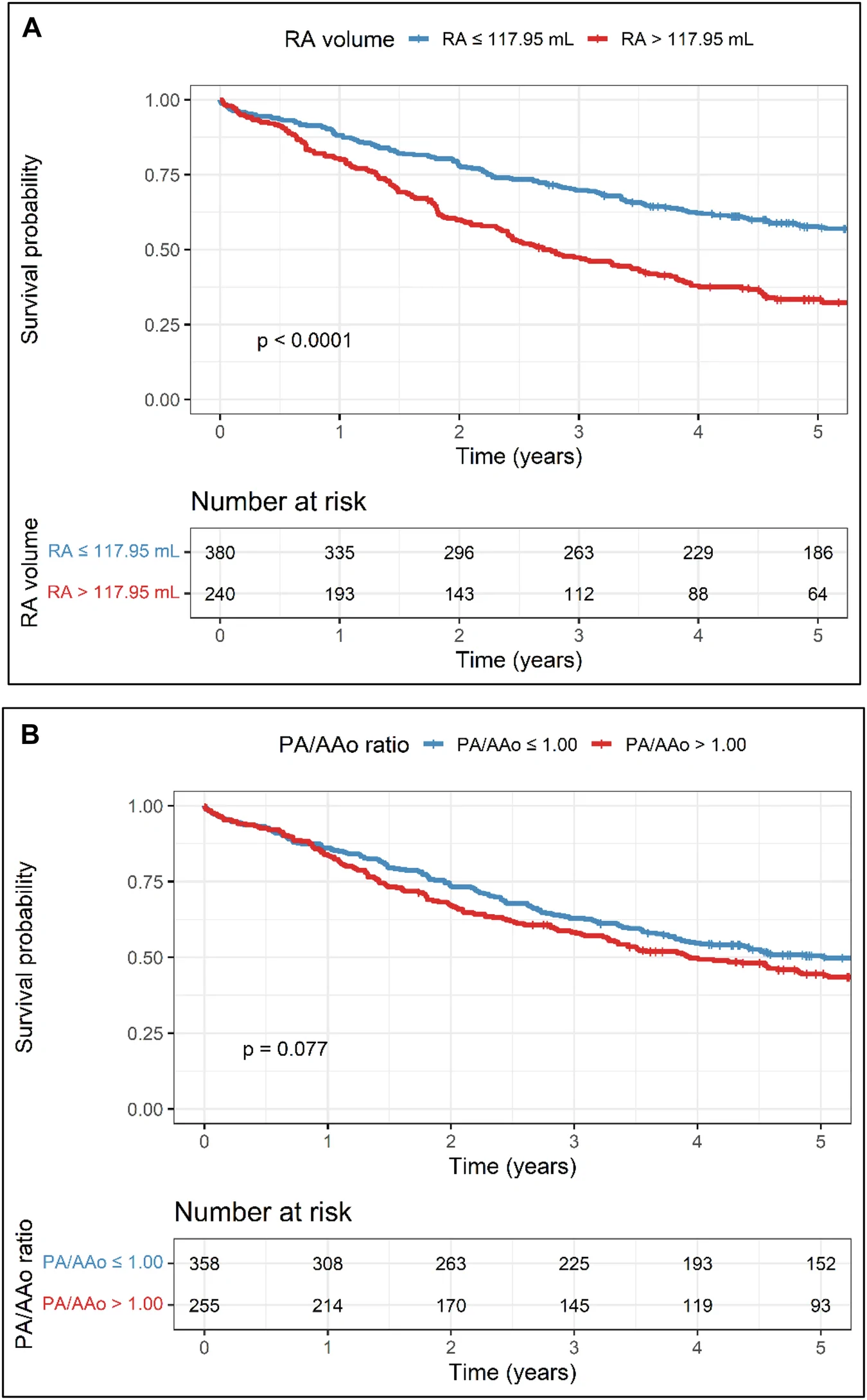
Kaplan-Meier survival curves for 5-year all-cause mortality according to AI-derived cardiac structure volumes. **A** Right atrial (RA) volume and (**B**) pulmonary artery (PA) to ascending aorta ratio. Blue lines represent low volume, and red lines represent high volume

RA volume above 117.95 mL was independently associated with increased mortality (HR 1.611, 95% CI: 1.298–1.999; *p* < 0.001). Similarly, a PA/AAo ratio > 1.0 demonstrated a strong independent association with mortality (HR 1.361, 95% CI: 1.094–1.694; *p* = 0.006), consistent with its recognised clinical relevance (Table 4 and Fig. 2).

**Table 4:** The association between the volumes and ratios of the various cardiac structures and mortality as categorised by Youden index followed by multivariable Cox regression analysis (adjusted for age, sex, and PH subgroups)

Segmented structure	Volume	HR	95% CI	*p*-value
RA	≤ 117.95 mL	1.611	1.298–1.999	< 0.001
	> 117.95 mL			
PA/AAo	≤ 1.00	1.356	1.090–1.686	0.006
	> 1.00			
RCA calcification	≤ 0.01 mL	1.533	1.240–1.895	< 0.001
	> 0.01 mL			
Mainstem + LAD calcification	≤ 0.01 mL	2.114	1.632–2.739	< 0.001
	> 0.01 mL			
LCX calcification	≤ 0.01 mL	1.465	1.158–1.855	< 0.001
	> 0.01 mL			
Total calcification	≤ 0.015 mL	2.026	1.554–2.641	< 0.001
	> 0.015 mL			

*RA* right atrium, *PA* pulmonary artery, *AAo* ascending aorta, *HR* hazard ratio, *CI* confidence interval, *mL* millilitre, *RCA* right coronary artery, *LAD* left anterior descending artery, *LCX* left circumflex artery

**Fig. 3:**
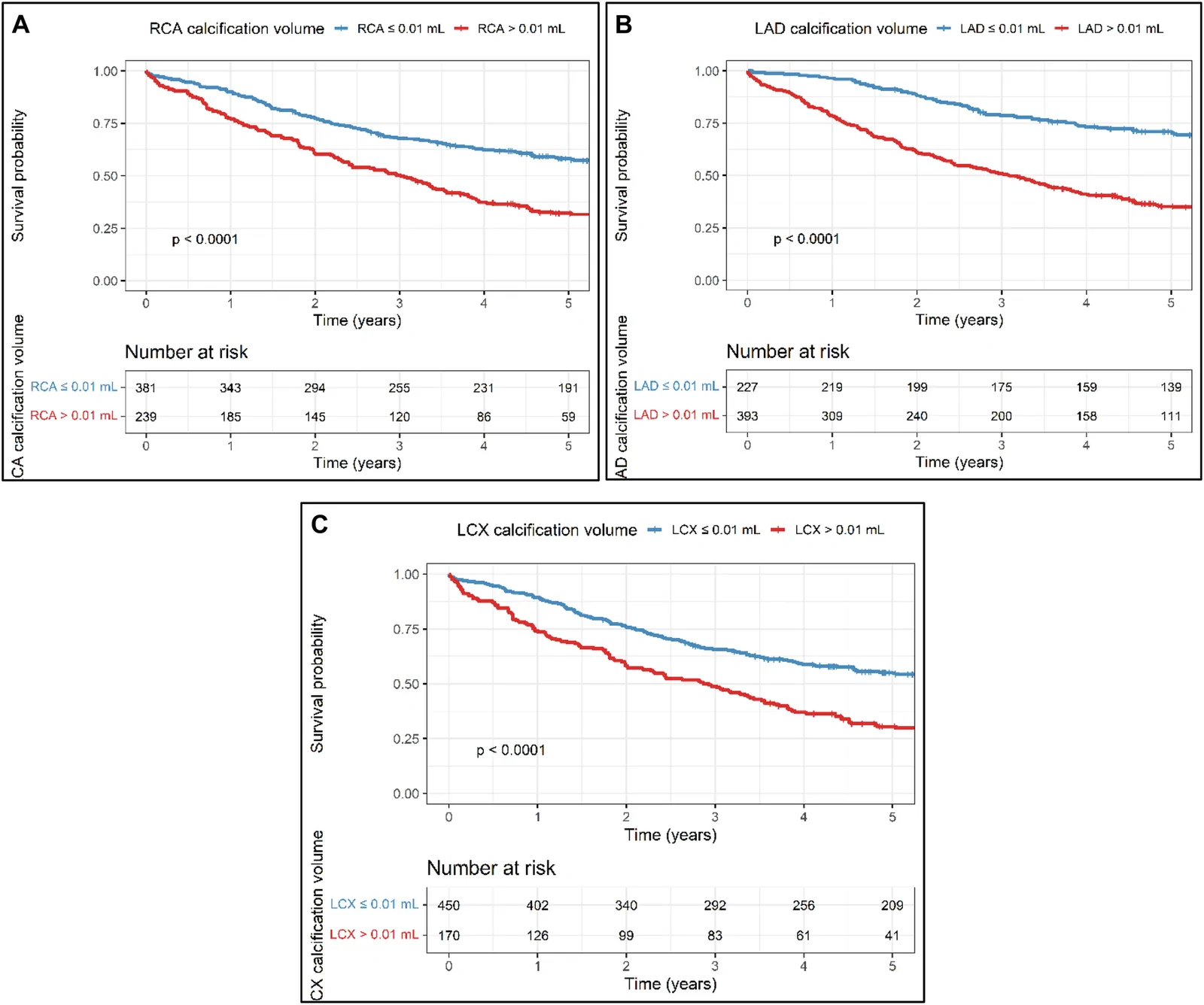
Kaplan–Meier survival curves for 5-year all-cause mortality according to AI-derived cardiac structure volumes (**A**) right coronary artery calcification volume, (**B**) mainstem and left anterior descending artery calcification volume, and (**C**) left circumflex artery calcification volume. Blue lines represent low volume, and red lines represent high volume

Coronary artery calcification remained a robust predictor. RCA calcification > 0.01 mL was associated with increased mortality (HR 1.533, 95% CI: 1.240–1.895; *p* < 0.001), as was mainstem and LAD calcification > 0.01 mL (HR 2.114, 95% CI: 1.632–2.739; *p* < 0.001), and LCX calcification > 0.01 mL (HR 1.465, 95% CI: 1.158–1.855; *p* < 0.001) (Table 4 and Fig. 3).

### Prognostic significance of AI-derived imaging variables after adjustment for REVEAL in PAH

LA and RA volumes were independently associated with increased mortality in PAH (LA: HR 1.046, 95% CI: 1.008–1.086, *p* = 0.016; RA: HR 1.039, 95% CI: 1.018–1.061, *p* < 0.001), whereas ventricles, AAo, PA, DAo, and PA/AAo > 1 were not significant.

Coronary artery calcification showed the strongest associations with mortality, including RCA (HR 1.616, 95% CI: 1.210–2.159, *p* = 0.001), mainstem+LAD (HR 2.829, 95% CI: 2.012–3.977, *p* < 0.001), and LCX (HR 1.662, 95% CI: 1.228–2.248, *p* < 0.001) (Table 5).

**Table 5:** Multivariable Cox regression analysis of AI-derived structure volumes and mortality in PAH (adjusted for REVEAL 2.0)

Structure	HR^+^	95% CI	*p*-value	Sig
Ventricles	0.998	0.987–1.009	0.660	
LA	1.046	1.008–1.086	0.016	*
RA	1.039	1.018–1.061	< 0.001	*
AAo	1.022	0.987–1.059	0.220	
PA	1.012	0.985–1.039	0.389	
DAo	1.032	0.991–1.074	0.128	
PA/AAo > 1	1.104	0.824–1.480	0.507	
RCA calcification	1.616	1.210–2.159	0.001	*
Mainstem + LAD calcification	2.829	2.012–3.977	< 0.001	*
LCX calcification	1.662	1.228–2.248	< 0.001	*
Calcification in any coronary artery	2.642	1.851–3.771	< 0.001	*
Calcification in all coronary arteries	1.621	1.173–2.241	0.003	*

*LA* left atrium, *RA* right atrium, *AAo* ascending aorta, *PA* pulmonary artery, *DAo* descending aorta, *RCA* right coronary artery, *LAD* left anterior descending artery, *LCX* left circumflex artery, *HR* hazard ratio, *CI* confidence interval

^*^ Significant. + HRs indicate hazard per 10-mL increase in AI-derived volume from non-contrast CT

## Discussion

Given that survival differences between PH subgroups have previously been reported from the ASPIRE Registry [21], the present study focused on the prognostic value of AI-derived cardiovascular structures and coronary artery calcification volumetric measurements in PH using non-gated, non-contrast CT scans, including their association with mortality and risk stratification. Several AI-derived volumetric measures of the cardiovascular structures and coronary artery calcification were significant predictors of mortality. RA volume and coronary artery calcification emerged as significant predictors in both univariable and multivariable analyses, while PA/AAo > 1 showed a robust association with mortality in multivariable analysis. When adjusted for the established risk score (i.e., REVEAL), RA and coronary calcifications remained significant predictors of mortality. Taking our study limitations into account, these findings indicate that integrating right heart morphology and coronary calcification may provide adjunctive prognostic value to established risk scores, supporting a more comprehensive, imaging-based approach to risk stratification.

While many prior studies have demonstrated that RA dilatation is associated with adverse outcomes, most have relied on ultrasound or magnetic resonance imaging rather than non-contrast CT [25, 26]. RA size and function have consistently been shown to correlate with mortality, clinical worsening, and disease severity in PH [9, 10, 25, 27]. In CT, a recent study in idiopathic PAH patients showed that RA dilatation was associated with adverse outcomes (HR 1.01, *p* = 0.02) [28]. In this context, our findings extend the literature by demonstrating that AI‑derived RA volume independently predicted mortality, with each 10‑mL increase conferring a 4% higher risk, and RA volumes > 117.95 mL associated with a 61% higher risk of mortality. Even after adjustment for the REVEAL score [22], each 10‑mL increase in RA volume was associated with a 2.6% higher risk of mortality. These findings support RA enlargement as a marker of right-sided pressure and volume overload and suggest RA dilatation as an additional biomarker for PH risk stratification [11, 25–30].

Prior CT studies evaluating PA morphology in PH have demonstrated that PA enlargement correlates with higher mPAP measured by RHC and PA/AAo > 1 is associated with adverse outcomes [13, 31]. Consistent with these observations, our study shows that, although PA/AAo > 1 was not significantly associated with mortality in univariable analysis, it became significant after adjustment for demographic and PH subgroups. However, this association was no longer significant after accounting for the REVEAL score, suggesting that the prognostic contribution of PA/AAo may largely reflect underlying disease severity already captured by the established clinical and haemodynamic variables incorporated within the REVEAL risk model [22].

Although non-dedicated CT scans are not specifically performed for coronary artery assessment, radiologists should routinely evaluate coronary artery calcification, given its well-established role as an independent predictor of adverse outcomes [15, 16, 24]. It has been demonstrated to independently predict all-cause and cardiovascular mortality, with both its presence and burden correlating with substantially increased risk of adverse outcomes. Importantly, this association remains significant even after adjustment for age and other risk factors [15, 16]. Our findings align with prior recommendations highlighting the prognostic importance of coronary artery calcifications. In univariable analysis, calcification in the RCA, mainstem and LAD, and LCX were all associated with increased risk of death. Patients with calcification > 0.01 mL in the RCA, mainstem, LAD, and LCX had approximately 53%, 111%, and 47% higher risk of mortality, respectively. These associations remained independent even after adjustment for REVEAL [22], highlighting the complementary role of imaging markers in risk stratification, particularly for LAD calcification, suggesting it may be especially relevant for adverse outcomes [32].

At present, validated risk stratification tools such as the REVEAL [22] score are established primarily for PAH patients [1]. To the best of our knowledge, there is no validated risk score applicable across all PH groups, and the prognostic assessment for non-PAH PH remains dependent upon clinical assessment, imaging findings, and haemodynamic parameters. In the context of the rising global prevalence of PH [3–5], and the increasing number of patients managed within specialist services in the UK [6, 7], this exploratory study has several clinical implications. First, AI-derived volumetric quantification of cardiothoracic structures and coronary artery calcification on CT may provide objective and reproducible biomarkers that can enhance risk stratification, consistent with recent studies [1, 20, 24, 27, 33]. As these scans are frequently performed for a variety of indications, automated extraction from existing scans may add incremental value without additional radiation [1, 24, 27].

A key strength of this study is the use of a well-curated retrospective CT dataset comprising a high proportion of patients with PH (76%), of whom 63% died. Coronary artery calcification was frequently observed, with involvement of the RCA (45%), mainstem and LAD (67%), and LCX (35%), reflecting the substantial burden of cardiovascular comorbidity within the cohort.

While this preliminary study demonstrates the potential prognostic value of AI-derived cardiovascular and coronary artery calcification volumes in PH patients, a number of limitations merit consideration. First, the study was retrospective, and the impact of the pipeline on clinical practice was not assessed. Second, all patients were recruited from a single centre, which may limit the generalisability of the findings. Moreover, the cohort was predominantly composed of White British patients, thereby restricting the ethnic diversity of the study population. Furthermore, most scans were performed using scanners manufactured by GE Healthcare, which may limit the applicability of the results across different imaging vendors. In addition, the sample size was not determined by an a priori power calculation. Finally, the observed mean survival time was 1659 ± 1207 days, reflecting substantial variability in follow-up duration. The study included scans acquired between 2006 and 2020 across multiple scanner platforms and reconstruction settings. Although all met predefined quality criteria, variation in acquisition parameters may have influenced volumetric measurements. Subsequent studies should seek to validate these findings using scans from multiple centres and a variety of scanner systems to enhance generalisability. Future studies should assess the prognostic value of these AI-derived volumes in more ethnically diverse populations and their impact on clinical practice to ensure reliability and generalisability.

## Conclusion

AI-derived volumetric measurements of the RA, and coronary artery calcifications in RCA, mainstem, LAD, and LCX on routine non-gated, non-contrast CT independently predict mortality in PH and demonstrate an independent and significant prognostic value even after adjustment for established risk scores. Although further work is required, these findings suggest that incorporating AI-derived imaging variables into established clinical risk score assessment may provide complementary information for risk stratification and improve prognostic assessment in PH patients.

## Data Availability

De-identified data within the ASPIRE registry can be made available for analysis to researchers who provide a methodologically sound proposal which fits within the aims of the ASPIRE registry. The ASPIRE data management committee will assess each proposal and decide within 3 months after submission. A data access agreement signed by the lead researcher and a data sharing agreement between Sheffield Teaching Hospitals and the recipient institution will be required before data is shared. Information and application forms can be found at https://bit.ly/aspire-registry. The trained model can be made available upon reasonable request for research purposes by non-commercial entities. Requests should be directed to the corresponding author.

## Abbreviations

AI: Artificial intelligence
ASPIRE: Assessing the spectrum of pulmonary hypertension identified at a referral centre
CT: Computed tomography
CTEPH: Chronic thromboembolic pulmonary hypertension
DAo: Descending aorta
DL: Deep learning
HRs: Hazard ratios
LA: Left atrium
LCX: Left circumflex coronary artery
mPAP: Mean pulmonary arterial pressure
PA: Pulmonary artery
PAH: Pulmonary arterial hypertension
PH: Pulmonary hypertension
PH-LHD: PH due to left heart disease
RA: Right atrium
RCA: Right coronary artery
RHC: Right heart catheterisation
REVEAL: Registry to evaluate early and long-term PAH disease management
RV: Right ventricular
SD: Standard deviation
